# Standardized quantification of biofilm in a novel rabbit model of periprosthetic joint infection

**DOI:** 10.5194/jbji-7-91-2022

**Published:** 2022-04-20

**Authors:** Anabelle Visperas, Daniel Santana, Minseon Ju, Nathalie B. Milbrandt, Yu Hsin Tsai, Sameera Wickramasinghe, Alison K. Klika, Nicolas S. Piuzzi, Anna Cristina S. Samia, Carlos A. Higuera-Rueda

**Affiliations:** 1 Department of Orthopaedic Surgery, Cleveland Clinic, Cleveland, OH, USA; 2 Cleveland Clinic Lerner College of Medicine, Case Western Reserve University, Cleveland, OH, USA; 3 Department of Chemistry, Case Western Reserve University, Cleveland, OH, USA; 4 Department of Orthopaedic Surgery, Cleveland Clinic Florida, Weston, FL, USA

## Abstract

Periprosthetic joint infection (PJI) is one of the most devastating complications of total joint arthroplasty. The underlying pathogenesis involves the formation of bacterial biofilm that protects the pathogen from the host immune response and antibiotics, making eradication difficult. The aim of this study was to develop a rabbit model of knee PJI that would allow reliable biofilm quantification and permit the study of treatments for PJI. In this work, New Zealand white rabbits ( n=19 ) underwent knee joint arthrotomy, titanium tibial implant insertion, and inoculation with Xen36 (bioluminescent *Staphylococcus aureus*) or a saline control after capsule closure. Biofilm was quantified via scanning electron microscopy (SEM) of the tibial explant 14 d after inoculation ( n=3 noninfected, n=2 infected). Rabbits underwent debridement, antibiotics, and implant retention (DAIR) ( n=6 ) or sham surgery ( n=2 noninfected, n=6 infected) 14 d after inoculation, and they were sacrificed 14 d post-treatment. Tibial explant and periprosthetic tissues were examined for infection. Laboratory assays supported bacterial infection in infected animals. No differences in weight or C-reactive protein (CRP) were detected after DAIR compared to sham treatment. Biofilm coverage was significantly decreased with DAIR treatment when compared with sham treatment (61.4 % vs. 90.1 %, p<0 .0011) and was absent in noninfected control explants. In summary, we have developed an experimental rabbit hemiarthroplasty knee PJI model with bacterial infection that reliably produces quantifiable biofilm and provides an opportunity to introduce treatments at 14 d. This model may be used to better understand the pathogenesis of this condition and to measure treatment strategies for PJI.

## 1 Introduction

Periprosthetic joint infection (PJI) is a devastating complication of total joint arthroplasty. Reported consequences of PJI include limited joint function, mobility, and a 5-year mortality rate of 26 %, similar to common cancers (Zmistowski and Parvizi, 2013; Kapadia et al., 2016). PJI is estimated to cost over USD 1.6 billion in the US with a case load of 70 000 revisions (Kurtz et al., 2012), and this is projected to increase by 68 %–176 % for knees and hips by 2030 (Schwartz et al., 2020). While treatment options are available, including irrigation and debridement (I&D), antibiotics, and one- and two-stage revision, the high treatment failure rate of 30 %–50 % (Sabry et al., 2014; Li et al., 2018) warrants further investigation into treatment strategies for effective infection eradication.

The underlying pathogenesis of PJI involves the formation of bacterial biofilm that protects the bacteria from both the host immune response and antibiotics, making it difficult to eradicate (Høiby et al., 2010). As this complexity will affect clinical decisions for diagnosis and treatment, models need to be clinically representative of the environment around the joint prostheses. Establishing an animal model with methods that build upon current animal models of PJI and that can introduce opportunities to test treatments with quantifiable readouts for both bacteria and biofilm is critical.

Animal models of PJI have utilized a variety of implants, ranging from simple wires to weight-bearing proximal tibia implants (Pribaz et al., 2012; Craig et al., 2005; Zhai et al., 2014; Carli et al., 2017). While these more sophisticated designs are more promising, the limited articular space and synovial fluid availability in smaller models remains a challenge with respect to introducing and evaluating the efficacy of treatment solutions, especially when assessing biofilm.

The purpose of this study was to develop a quantifiable method to assess biofilm coverage with bacteria readout confirmation in a novel, reproducible, rabbit model of knee PJI with opportunities to introduce treatments.

## 2 Materials and methods

### 2.1 Animals

A total of 19 female New Zealand white rabbits (Charles River Laboratories, Wilmington, MA) were utilized (average preoperative weight of 3.5 kg ± 0.4 kg and approximate age of 16–18 weeks based on their weight at the start of study; Masoud et al., 1986). Females were chosen for these surgeries due to their larger size compared with males. It should be noted that no differences in the incidence or treatment success between sexes were detected in human PJI from a cohort of more than 1000 patients (Mironenko et al., 2021). Animals were housed individually with natural light–dark cycles and were allowed free access to food and water. The sample size was calculated based on the assumption of a 25 % difference in biofilm coverage (variance of 15 %) and scanning electron microscopy (SEM) with 80 % power between sham and DAIR treatment. Rabbits were randomized into groups by picking numbers out of a container. This study was approved by the Institutional Animal Care and Use Committee.

### 2.2 Bacteria preparation

Xen36, bioluminescent *Staphylococcus aureus* (ATCC 49525, American Type Culture Collection, Manassas, VA) was cultured overnight in kanamycin sulfate (200 µg mL ${}^{-1}$ ) Luria broth (LBK) with agitation at 200 rpm at 37 ${}^{\circ }$ C. Bacterial solutions were resuspended in saline at $5.0\times 10^{7}$ CFU mL ${}^{-1}$ , based on absorbance, and inoculated within 4 h of preparation.

### 2.3 Implant

**Figure 1:**
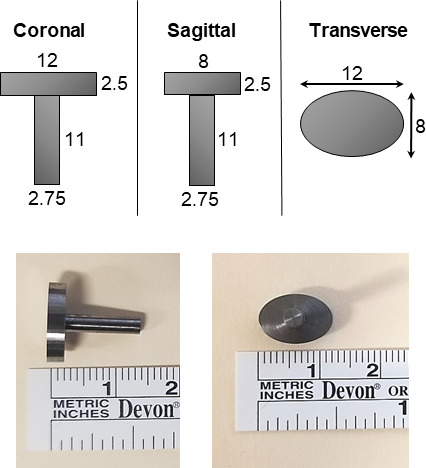
Custom-made titanium implant design and titanium tibial implant dimensions (in mm).

A computer numerical control (CNC) machined rabbit-sized tibial implant composed of titanium (Ti-6Al-4V) was custom-made and utilized (Biomedical Engineering Prototype Lab, Cleveland Clinic, Cleveland, OH). The head dimensions of the implant were as follows: 12 mm major axis and 8 mm minor axis with 2.5 mm thickness. The intramedullary stem was 11 mm in length and 2.75 mm in diameter (Fig. 1). All finishes were machined finishes. The stem was aluminum oxide that was sandblasted to roughen the surface. Implants were steam sterilized at 173 ${}^{\circ }$ C for a full cycle.

### 2.4 Surgical procedure

**Figure 2:**
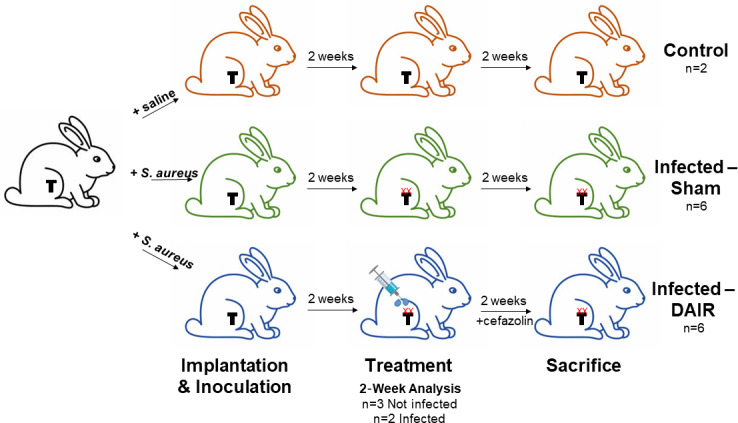
Experimental schematic: all rabbits were implanted with a titanium press-fitted or cemented tibial implant; control and infected rabbits received an intra-articular injection of saline or Xen36, respectively, after capsule closure; 2 weeks later, when rabbits had a productive infection with biofilm (denoted with XX), they underwent either sham treatment or debridement, antibiotics, and implant retention (DAIR) treatment where they received an irrigation and debridement (I&D) and cefazolin antibiotics for 2 weeks; finally, rabbits were sacrificed 2 weeks post-treatment for post-mortem analysis of bacterial biofilm formation and bacterial burden.

Figure 2 presents the experimental schematic. For detailed surgical procedures, the reader is referred to Method S1 in the Supplement.

*Index surgery (implantation and inoculation)*. After sedation and site preparation, a 4.5 cm parapatellar incision was made into the right knee. The patella was dislocated laterally, and the anterior cruciate ligament (ACL) and menisci were resected. A bone saw was used to remove ∼ 2.5 mm of the articular cartilage and proximal epiphysis of the tibia. A burr was then used to create a hole in the medullary canal. The implant was press-fitted ( n=5 for 14 d analysis, n=7 for 28 d analysis) or cemented ( n=7 for 28 d analysis) into the proximal tibia (see the methods in the Supplement for a breakdown of groups). The joint capsule was closed with surgical knots using 3-0 monofilament nylon. Rabbits were inoculated intra-articularly with a 25G needle with either $5\times 10^{6}$ CFU Xen36 in 100 µL saline or a saline control after capsule closure. The skin was closed with a running stitch using 3–0 monofilament nylon (Fig. S1 in the Supplement). Average surgery time was 25.5 min ± 7 min.

*For treatment surgery* ( + *14 d*). This time point was chosen due to consistent biofilm presence on the implant and owing to the relevance of this time point in treatment scenarios in humans. There is no evidence-based timeline for PJI treatment, as the actual history of infection is unknown until symptoms are presented. In humans, with implant retention, treatment is more likely to fail when symptom duration/primary implantation is beyond 4 weeks (Elkins et al., 2019). Synovial fluid and periprosthetic tissue were sampled. For the debridement, antibiotics, implant retention (DAIR) group, necrotic and purulent tissues were removed, lavaged with saline, mechanically brushed with an interdental brush, lavaged again with saline, and the incision was closed in a similar fashion to the index surgery. Rabbits received cefazolin twice a day for 2 weeks (20 mg kg ${}^{-1}$ ). For the sham group, samples were obtained and the incision was closed. Average surgery time for the sham treatment was 11.5 min ± 2 min, and average surgery time for the DAIR treatment was 19.5 min ± 2 min.

*For sacrifice* ( + *28 d*). Rabbits were euthanized with pentobarbital. Synovial fluid, periprosthetic tissue, and the implant were collected. All samples were blinded to the treatment arm prior to analysis.

### 2.5 Cell assays

The Musculoskeletal Infection Society (MSIS) criteria includes cultures to diagnose PJI (Parvizi and Gehrke, 2014). Tissue samples were suspended in 3 mL of LBK and vortexed (Vortex-Genie, Fisher Scientific, Waltham, MA) for 1 min, followed by sonication for 5 min (Branson 2800 sonication bath) at 40 kHz and a power density of 0.22 W cm ${}^{-2}$ . Each sample was incubated for 2 weeks with agitation (200 rpm) at 37 ${}^{\circ }$ C and plated overnight at 37 ${}^{\circ }$ C. For bioluminescence (BLI), plates were imaged using a GloMax multi-detection reader system (Promega, Madison, WI).

### 2.6 Laboratory test

Peripheral blood was taken prior to each surgery and at sacrifice to assess other laboratory tests typically included in the workup to diagnose human PJI as per the MSIS criteria (Parvizi and Gehrke, 2014). Blood was collected from the auricular vein and spun down at 3700 rpm for 15 min. Plasma was stored at - 80 ${}^{\circ }$ C until use. Rabbit C-reactive protein (CRP) ELISA (Enzyme- Linked Immunosorbent Assay; Immunology Consultants Laboratory, Portland, OR) was plated in duplicates as per the manufacturer's protocol.

### 2.7 Histology

Synovium tissue samples were fixed for 24 h in 10 % buffered formalin, paraffin-embedded, and stained with Harris modified hemotoxylin and eosin (H&E). Images were taken at 10× objective magnification using an Olympus CKX41 inverted phase contrast microscope (Olympus America, Center Valley, PA). Slides were blinded and reviewed by two individual reviewers using the criteria for inflammation shown in Table S1 in the Supplement (taken from Orange et al., 2018). Scores were combined from each reviewer for a total of 10–12 data points per group per parameter, and the Kappa statistic was calculated to assess inter-rater agreement.

### 2.8 SEM processing and image analysis

For detailed procedures, the reader is referred to Methods S3 in the Supplement.

**Figure 3:**
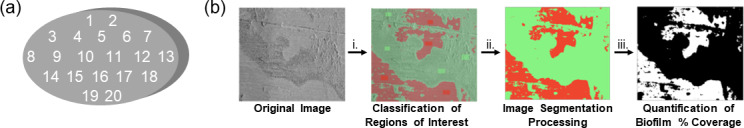
SEM analysis showing **(a)** a schematic diagram depicting the regions of interest used in the SEM image analysis of the implants and **(b)** an SEM image processing using the automated Trainable Weka Segmentation approach: (i) selection of training samples to classify regions of interest – biofilm-free surface (red) and biofilm-covered surface (green); (ii) image segmentation processing; and (iii) quantification of the percent biofilm coverage.

*Processing*. The explant was fixed with paraformaldehyde and then dehydrated in increasing ethanol concentrations. Samples were vacuum-dried overnight, sputter-coated with 15 nm of gold, and imaged using a Zeiss SIGMA VP field emission SEM (White Plains, NY). A custom script was used to automate the SEM stage and image capture. Twenty predetermined image locations were collected at 1500× magnification at the top of the implant (Fig. 3a). Image sampling covered 0.5 % of the top of implant area. It should be noted that representative images were collected from noninfected implants only.

*Analysis*. The Trainable Weka Segmentation plugin in Fiji (distribution of ImageJ, National Institutes of Health, Bethesda, MD) was used for analysis. Ten regions of interest (ROIs) were selected to identify biofilm-absent and biofilm-present regions on each image for segmentation. In total, 25 images were used to train the classifier, and this classifier was used to calculate the percent biofilm coverage on all subsequent images (Fig. 3b).

### 2.9 Statistics

For continuous variables, a Student t test or one-way ANOVA with a Tukey post hoc test was carried out, and for categorical variables, a Fisher exact test was calculated, all using GraphPad Prism 8.0 (San Diego, CA). All data are depicted as the mean ± SE (standard error of the mean). Inter-rater agreement was assessed using the kappa statistic in GraphPad QuickCalcs (San Diego, CA).

## 3 Results

### 3.1 Gross assessment

**Figure 4:**
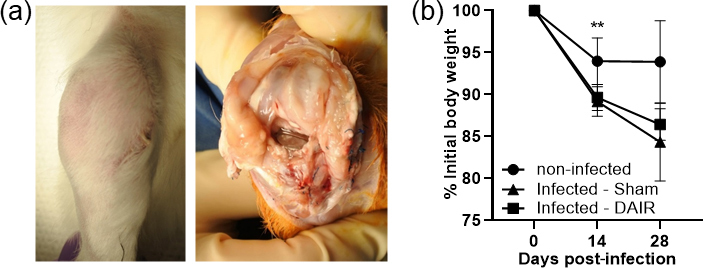
Gross findings and weight loss showing **(a)** representative gross images at 14 d post-infection with $5\times 10^{6}$ CFU of Xen36, and **(b)** the weight change in rabbits over time, plotted as the percent weight change from the initial weight. In panel **(b)**, n=4 for the control treatment and n=6 for infected treatments at 0 d and 14 d, and n=3 for the control treatment and n=6 for infected treatments at 28 d ( ${}^{**}$ p=0 .0023 at 14 d).

All rabbits survived the index surgery without complications. No disturbed wound healing or clinical signs of systemic illness (e.g., decreased appetite, behavior changes, body temperature) were seen throughout the experiment. Rabbits began to bear weight by postoperative day (POD) 1 and started using the limb by POD 5–7. It should be noted that one control rabbit had a dislocated knee (surgical side) due to a medial collateral ligament tear 2 days after index surgery. No signs of any additional pain were observed, and the animal remained under veterinarian care during the study. Implant instability was not obvious in control or infected animals via physical inspection and radiography. Gross findings after 2 weeks revealed mild outgrowths of the synovial membrane seen in the knee of infected animals (Fig. 4a). The knees of infected rabbits were mildly inflamed, and the joint fluid was thicker with purulence, whereas the control animals were negative for signs of infection and had clear synovial fluid.

In total, 14 of the 16 rabbits survived the treatment surgery with no major complications. Two rabbits died at the 14 d time point during anesthesia and were used for the 2-week analysis. It should be noted that rabbits have a higher incidence of peri-anaesthetic mortality with no underlying conditions compared with other animals (Lee et al., 2018). Overall, control animals maintained their weight throughout the experimental timeline, whereas infected rabbits lost weight over the course of infection, although only trending at 28 d (Fig. 4b). Treatment with DAIR did not affect weight loss. One rabbit randomized to the sham group received cefazolin (IM, 20 mg kg ${}^{-1}$ , twice a day) from day 25 to 28 due to surgical site infection that manifested with purulence from the wound.

### 3.2 Bacterial infection confirmation – laboratory and histological assessments

**Figure 5:**
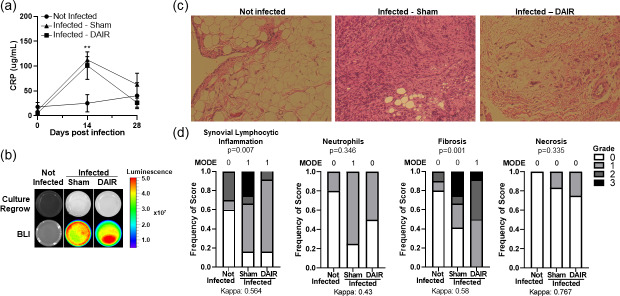
Panels **(a–d)** show readouts of bacterial infection. **(a)** Peripheral blood was collected as a baseline and then at 14 and 28 d after infection, and it was analyzed for CRP levels by ELISA. For the baseline and 14 d, n=5 –6, and for 28 d, n=3 –6 for the two experimental treatments ( ${}^{**}$ p=0 .0022 at 14 d). Panel **(b)** shows a representative figure of 2-week culture regrowth and BLI from tissues taken 28 d after infection. Panel **(c)** presents representative images of Harris modified H&E-stained synovium tissue from rabbits infected 28 d prior. A 10× magnification was utilized, and n=5 –6 per group for the two experiments. Panel **(d)** shows the scores of synovitis-related markers of inflammation from two individual blinded reviewers ( n=5 –6 per group for the two experiments).

CRP, one of the nonspecific markers of infection, was significantly elevated at 14 d after infection in infected animals ( p=0 .0022; Fig. 5a). By 28 d, CRP levels had decreased in infected samples.

Tissue samples were used for culture regrowth experiments to confirm the growth of bioluminescent bacteria that was inoculated during index surgery. Luminescence was apparent after 2 weeks of culture regrowth via BLI (Fig. 5b).

Differences in synovium histology between noninfected and infected samples were apparent (Fig. 5c). Some scores of inflammation-related parameters, including synovial lymphocyte inflammation ( p=0 .007), neutrophils ( p=0 .346), fibrosis ( p=0 .001), and necrosis ( p=0 .335), were significant, with inter-rater agreement kappa scores between moderate and substantial agreement (Fig. 5d).

### 3.3 Bacterial biofilm assessment – scanning electron microscopy

**Figure 6:**
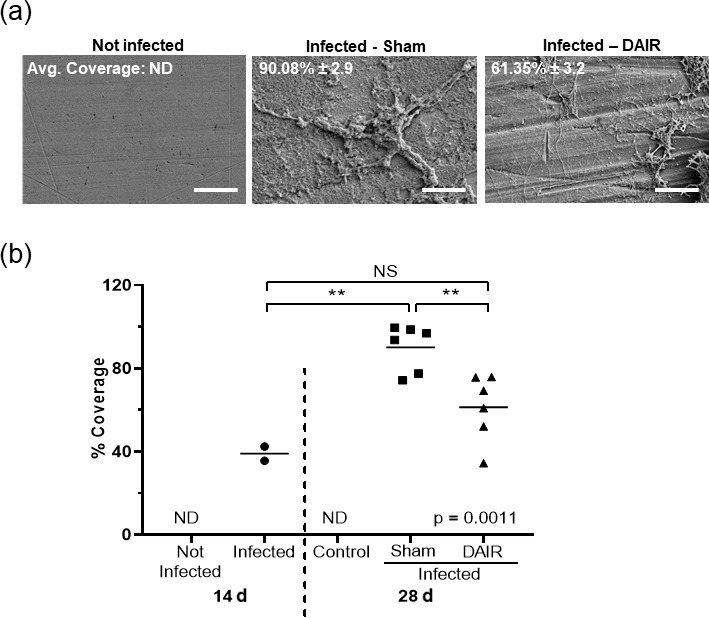
Panels **(a)** and **(b)** present the SEM analysis of biofilm coverage. Panel **(a)** shows representative SEM images of biofilm coverage at 28 d at 1500× magnification (scale bar 40 µM ). The average coverage is shown as the mean ± SE. Panel **(b)** presents plots depicting the percent coverage at 14 and 28 d after infection. Each dot is the average of the 20 points analyzed on each implant ( n=2 –3 at 14 d; n=2 for the control and n=6 for sham and DAIR at 28 d from the two experimental treatments). ND: not determined; NS: not significant. The ANOVA p value was p=0 .0011, and ${}^{**}$ denotes p<0 .01.

While bacterial infection was confirmed using multiple readouts, biofilm coverage is still a major contributor to the difficulty in treating infections. Methods to systematically quantify biofilm burden, especially to assess the effectiveness of treatments, have not be addressed. Biofilm coverage was assessed via SEM analysis at 14 and 28 d after infection. SEM images showed that implants obtained from the infected animals had grown *S. aureus* biofilm on the surface of the implants after 14 d (Fig. 6b). As biofilm was consistently present on the implants of the infected animals analyzed, we concluded that biofilm formation was evident at 14 d and that this was a suitable time point for treatment intervention, including DAIR.

At 28 d, the implants isolated from infected rabbits were positive for biofilm, whereas the implants from the control animals showed no sign of bacterial biofilm (Fig. 6a). There was a significant increase in biofilm coverage from 14 to 28 d for sham treatment ( p=0 .0017, Fig. 6b). DAIR significantly decreased biofilm coverage from 90.08 % to 61.35 % ( p=0 .0083, Fig. 6b).

## 4 Discussion

In order to measure the effectiveness of treatments against PJI, two major criteria are missing from the current literature: (1) a method to objectively quantify biofilm and (2) a representative PJI model that includes an opportunity for interventions to be introduced and tested.

The majority studies in the literature have focused on infection readouts, like CFUs and cultures, as a marker of successful eradication (Sosa et al., 2020; Breyne et al., 2017; Sultana et al., 2015), but they do not account for biofilm that may be left behind as a reservoir for bacteria. Others have descriptively assessed or have picked sites to quantify biofilm burden on implants via SEM (Vyas et al., 2016; Gomes and Mergulhão, 2017); however, these methods provide a nominal readout or a glimpse into the infection status. Therefore, we have developed a quantitative readout of biofilm that is backed by commonly used laboratory and histology tests that support PJI diagnosis. Indeed, with biofilm quantification, we were able to assess the effectiveness of the commonly used DAIR treatment with respect to decreasing biofilm burden at levels possibly indicative of failure in human infection (Kim et al., 2019; Xu et al., 2020).

Four standardized animal model criteria have been suggested to be clinically representative of PJI and necessary to test treatment strategies: (1) animal must share similarities to humans in regards to the immune and musculoskeletal system; (2) relevant weight-bearing implant material that reproduces the periprosthetic environment must be chosen; (3) a clinically relevant bacterial strain must be used; and (4) appropriate readouts to measure biofilm, bacteria, and immune response are required (Carli et al., 2016). Many studies have touched upon some of these suggestions, but our presented PJI model has improved upon previous work in the field and has begun to incorporate relevant treatments for PJI, including DAIR. Rabbits were chosen for this model due to their robustness to multiple surgeries, their similarities to human bone, and their synovial fluid volume range from 50 to 2200 µL (McCarty et al., 2011), which is useful in testing local treatment strategies compared with mice that have volumes from 2 to 5 µL (Seifer et al., 2008). A weight-bearing titanium hemiarthroplasty design was used to create interactions with two distinct spaces: the hypovascular immune-privileged articular space and the hypercellular intramedullary space that promotes the host immune system and bacterial interactions in a clinically relevant manner (Yang et al., 2014; Carli et al., 2017; Wijeyekoon et al., 2004; Liu and Tay, 2001; Jie et al., 2019). We also included cemented implants in one cohort of this study to increase implant stability and for clinical relevance, and data were consistent between experiments regardless of cementing. This model used a clinically relevant bacterial strain, *S. aureus*, which accounts for 38 % of knee and hip PJIs (Tande and Patel, 2014). For bacterial load, $5\times 10^{6}$ CFU proved to be an optimal concentration for reproducible infection and the presence of biofilm on the implant with no major side effects. In our studies, rabbits with $5\times 10^{7}$ CFU had a robust infection that was difficult to effectively manage, whereas concentrations below $5\times 10^{5}$ CFU inconsistently produced infections (Belmatoug et al., 1996). This work includes multiple readouts for infection, including SEM, cultures, tissue histology, and CRP. It should be noted that significant increases in inflammation and fibrosis with infection were observed by histology, but a post hoc analysis with a small sample size could not establish whether sham and DAIR groups were different. These readouts were congruent with the definition of PJI according to the MSIS criteria, although exact criteria in rabbits are unknown.

There were several limitations to our study. First, while CRP was completed, additional laboratory tests included in the MSIS criteria (Parvizi et al., 2011) , e.g., erythrocyte sedimentation rate, synovial fluid cell count, may be integrated into future iterations of this model, although they were outside the scope of the current study. Second, this hemiarthroplasty model does not include a true metal-on-polyethylene articulation commonly used in human total knee arthroplasty (TKA) patients. A noninfected rabbit study created an ultrahigh-molecular-weight polyethylene tibial and cobalt–chromium alloy femoral component; however, it must be cautioned that a rabbit joint may not be large enough to accommodate multiple components, as these rabbits developed gait issues with joint overstuffing (study reviewed in Jie et al., 2019). Nevertheless, this hemiarthroplasty does produce a biofilm infection on a weight-bearing surface and is, therefore, useful in understanding PJI biology. Third, BLI was used for in vitro bacterial infection confirmation only. Imaging in vivo would be a valuable addition, but detecting bioluminescence is currently limited to smaller animals due to signal attenuation through tissue (Xu et al., 2016). Implant imaging was also below the limit of detection, which was most likely due to the low levels of metabolically active bacteria within the biofilm. Fourth, while we did see a 30 % decrease in biofilm coverage with DAIR treatment, it remains to be determined if this is clinically significant or supports the need for dual treatments. The surgical time points in this study are also a proof-of-concept which show that rabbits were able to withstand multiple invasive surgeries and a robust infection for 28 d and that treatments can be introduced at the 14 d time point. Indeed, in future iterations of this model, other treatment options will be introduced, including antibiotic treatments, alternative irrigation solutions, and novel biofilm-disrupting agents.

## 5 Conclusions

This experimental rabbit knee PJI model shows the reliable establishment of infection with consistent biofilm formation on implants, incorporates a clinically relevant I&D procedure, and allows for comparison of different biofilm treatment approaches. This model has the potential to be a tool to test local prevention and therapeutic strategies for PJI, with a special focus on the treatment of biofilm through a standardized quantitative approach.

## Supplement

10.5194/jbji-7-91-2022-supplement The supplement related to this article is available online at: https://doi.org/10.5194/jbji-7-91-2022-supplement.

## Data Availability

No data sets were used in this study.
