# Supplementary material for: Standardized quantification of biofilm in a novel rabbit model of periprosthetic joint infection
Source: J Bone Jt Infect. 2022 Apr 20;7(2):91–9. doi: 10.5194/jbji-7-91-2022 (PMC9051660; doi:10.5194/jbji-7-91-2022)
Supplement: The supplement related to this article is available online at: https://doi.org/10.5194/jbji-7-91-2022-supplement. [file jbji-7-91-supplement.pdf]

Supplement of J. Bone Joint Infect., 7, 91–99, 2022
https://doi.org/10.5194/jbji-7-91-2022-supplement
© Author(s) 2022. CC BY 4.0 License.

Supplement of

Standardized quantification of biofilm in a novel rabbit model of periprosthetic joint infection

Anabelle Visperas et al.

Correspondence to: Alison K. Klika (klikaa@ccf.org)

The copyright of individual parts of the supplement might differ from the article licence.

Supplemental Methods

S1 Surgical procedure

S1.1 For all surgeries – Animal was intubated and a catheter was placed in the auricular vein for administration of maintenance fluids during surgery. Before sedation, rabbits were given oral meloxicam (0.3 mg kg^{-1}), intramuscular (IM) buprenorphine (0.05 mg kg^{-1}) and intravenous (IV) cefazolin (20 mg kg^{-1}). Surgery was carried out under general anesthesia induced by IM ketamine (35 mg kg^{-1}) and midazolam (3 mg kg^{-1}) and continuous inhalation of 1 % isoflurane. The lower extremity was shaved, sterilized with 70 % alcohol and 7.5 % povidone iodine solution, and draped. Subcutaneous (SQ) bupivacaine (0.2-0.4 mg kg^{-1}) at surgical site was used prior to skin incision. Post-operatively, rabbits were given oral meloxicam daily for two days and IM buprenorphine twice a day for three days and as needed post-operatively. Bandage was removed after 5-7 days.

S1.2 For index surgery (implantation and inoculation) – A 4.5 cm medial parapatellar incision was made in the right knee. Patella was dislocated laterally and anterior cruciate ligament (ACL) and menisci were resected. With use of a 29.5x7x0.38 mm long bone saw with Command 2 MicroElectric System instruments (Stryker Orthopaedics, Mahwah, NJ), the articular cartilage and proximal epiphysis of the tibia measuring ~2.5 mm thickness was removed while protecting the collateral ligaments and soft tissues (Figure S1a). A 2.3 mm tip burr (Stryker Orthopaedics, Mahwah, NJ) was used to create a hole in the medullary canal (Figure S1b). The implant was press-fit or cemented (Zimmer Biomet Bone Cement R, Warsaw, IN) into the proximal tibia (Figure S1c). After implantation, saline joint lavage, and hemostasis, joint capsule was closed with surgical knots using 3-0 monofilament nylon. Rabbits were inoculated intra-articularly using a

1mL syringe with a 25G needle with $5x10^6$ CFU *Xen36* in 100 µl saline or saline only after capsule closure. Skin was closed with a running stitch in layers with a 3-0 monofilament nylon. Fluoroscopy of all operative knees was obtained post-operatively to check the position of the implant (Figure S1d).

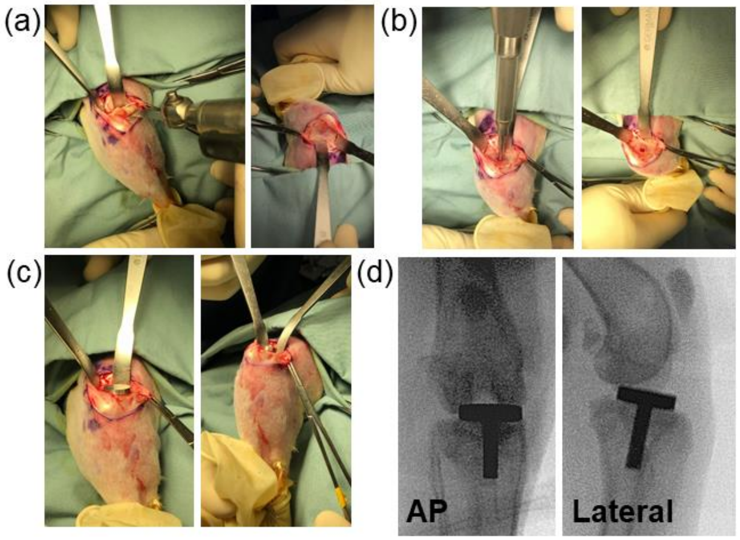

Supplemental Figure 1. Implant placement (a) 2.5mm of the tibial plateau was removed using a surgical saw. (b) After burr hole was introduced, (c) implant was press-fit or cemented into the tibia. (d) Radiograph of implantation into the right knee. AP = anterior/posterior view.

S1.3 For treatment surgery (+2-weeks): This treatment time point was chosen due to a consistently productive bacterial biofilm present on the surface of the implant and the relevance of this time point in treatment scenarios in humans. Upon opening, synovial fluid and tissue from femur, synovium, and tibia were collected for cultures and histology. Rabbits in the debridement, antibiotics, implant retention group (DAIR), underwent irrigation and debridement (I&D) where necrotic and purulent tissues were removed, lavage with 100 mL saline through a 25G needle, 1

minute mechanical brushing (simple interdental brushes, up&up Target brand, Minneapolis, MN) of the implant surfaces and soft tissues, lavage with 100 mL saline, and closed similar to index surgery. Rabbits received cefazolin twice a day for two weeks (20 mg kg^{-1}). For the sham group, synovial fluid and soft tissue samples were obtained and closed.

S1.4 For *sacrifice (+4-weeks):* Two weeks following treatment, rabbits were euthanized by intravenous (IV) injection of pentobarbital (100 mg kg^{-1}) after induction of general anesthesia via IM injection of ketamine (35 mg kg^{-1}) and midazolam (3 mg kg^{-1}). Synovial fluid and tissue were collected similarly to the two week timepoint. Implant was stored in media until analysis. All samples were blinded to treatment arm prior to analysis. Final groups of press-fit implants were N=5 at 14 day analysis (N=3 non-infected, N=2 infected), N=7 at 28 day analysis (N=2 non-infected, N=3 sham, N=2 DAIR); cemented N=7 (N=3 sham, N=4 DAIR).

S2 Histology

**Lymphocytic Inflammation**	
**0**	None, less than one perivascular aggregate in at least two fields
**1**	Mild, one perivascular aggregate in at least two fields
**2**	Moderate, more than two perivascular aggregates
**3**	Marked, widespread perivascular/interstitial aggregates
**Neutrophils**	
**0**	None
**1**	Present
**Fibrosis**	
**0**	None
**1**	Focal
**2**	Widespread
**3**	Band-like
**Necrosis**	
**0**	None
**1**	Necrosis present in less than 50% of sample
**2**	Necrosis present in more than 50% of sample

Supplemental Table 1. Histology scoring system for synovitis

S3 Scanning electron microscopy (SEM) processing and image analysis

S3.1 SEM Processing

After animal sacrifice, the metal explant was fixed with 4 % paraformaldehyde in phosphate-buffered saline (PBS) (Fisher Scientific, Hampton, NH) for 1 hour. Implants were rinsed three times with 0.1 M PBS solution, and subsequently dehydrated using an ethanol solution series containing 50 %, 70 %, 80 % and 100 % ethanol (for 10 min each step) (Sigma-Aldrich, St. Louis, MO). Samples were vacuum dried overnight and sputter coated with 15 nm of gold and analyzed using a Zeiss SIGMA VP-FESEM (White Plains, NY). To facilitate quantitative mapping of the total coverage of the biofilms on each implant, a custom script written in DigitalMicrograph software (Gatan Inc., Pleasanton, CA) was employed to automate the SEM stage and image capture. Twenty images were collected at 1,500 x magnification and 3 kV from the top of the implant, each image were set ~2 mm apart and were selected based on a statistically designed area distribution (Figure 3a). Image sampling covered 0.5 % of the total top of implant area. Of note, due to the absence of a bacterial infection on control implants, representative images only were collected for this group.

S2.2 SEM Image Analysis

Each SEM image was segmented using the Trainable Weka Segmentation plugin, a machine learning alogorithm, in Fiji (distribution of ImageJ, NIH, Bethesda, MD). Around 10 regions of interest (ROI) were selected to identify the non-biofilm or biofilm regions on each SEM

image. 25 images were used to train the classifier and the saved classifier could be applied for subsequent SEM images. The segmentation result was generated and analyzed by the percentage area coverage calculator on Fiji (Figure 3b). The following training setting features were chosen: Gaussian blur, Sobel filter, Hessian, Difference of Gaussians, Membrane projections, Variance, Mean, Minimum, Maximum, Median and Bilateral(Vyas et al., 2016).
